# Post-diagnosis serum insulin-like growth factors in relation to dietary and lifestyle changes in the Prostate testing for cancer and Treatment (ProtecT) trial

**DOI:** 10.1007/s10552-017-0910-2

**Published:** 2017-06-23

**Authors:** Vanessa Er, Kalina Biernacka, Andrew J. Simpkin, Richard M. Martin, Mona Jeffreys, Pauline Emmett, Rebecca Gilbert, Kerry N. L. Avery, Eleanor Walsh, Michael Davis, Jenny L. Donovan, David E. Neal, Freddie C. Hamdy, Jeff M. P. Holly, J. Athene Lane

**Affiliations:** 1 School of Social and Community Medicine, University of Bristol, Canynge Hall, 39,Whatley Road, Bristol, BS8 2PS UK; 2 National Institute for Health Research (NIHR) Bristol Nutrition Biomedical Research Unit, Level 3, University Hospitals Bristol Education & Research Centre, Upper Maudlin Street, Bristol, BS2 8AE UK; 3 IGFs and Metabolic Endocrinology Group, School of Clinical Sciences, University of Bristol, Learning and Research Building, Southmead Hospital, Bristol, BS10 5NB UK; 4 Medical Research Council Integrative Epidemiology Unit, Oakfield House, Oakfield Grove, Bristol, BS8 2BN UK; 5 Nuffield Department of Surgical Sciences, University of Oxford, John Radcliffe Hospital, Oxford, OX3 9DU UK

**Keywords:** Prostatic neoplasms, Diet, Lifestyle, Insulin-like growth factors, Post-diagnosis

## Abstract

**Purpose:**

The insulin-like growth factor (IGF) system is modifiable by diet and lifestyle, and has been linked to prostate cancer development and progression.

**Methods:**

We conducted a prospective cohort study of 621 men diagnosed with localized prostate cancer to investigate the associations of dietary and lifestyle changes with post-diagnosis circulating levels of IGF-I and IGFBP-3. We used analysis of covariance to estimate the associations, controlling for baseline IGF-I or IGFBP-3, respectively.

**Results:**

Mean IGF-I levels were 6.5% (95% CI −12.8, −0.3%, *p* = 0.04) lower in men who decreased their protein intake after diagnosis compared to men who did not change. Men who changed their fruit and vegetable intake had lower IGF-I levels compared to non-changers [Decreased intake: −10.1%, 95% CI −18.4, −1.8%, *p* = 0.02; Increased intake: −12.0%, 95% CI −18.4, −1.8%, *p* = 0.002]. IGFBP-3 was 14.6% (95% CI −24.5, −4.8%, *p* = 0.004) lower in men who achieved a healthy body mass index after diagnosis. Men who became inactive had 9.5% higher average IGF-I levels (95% CI 0.1, 18.9%, *p* = 0.05).

**Conclusions:**

Decreased protein intake and body mass index, and increased physical activity and fruit and vegetable intake, following a prostate cancer diagnosis were associated with reduced post-diagnosis serum IGF-I and IGFBP-3. Counterintuitively, reduced fruit and vegetable intake was also associated with reduced IGF-I, but with weak statistical support, possibly implicating chance. If confirmed in other studies, our findings may inform potential lifestyle interventions in prostate cancer. ProtecT was registered at International Standard Randomised Controlled Trial Registry, http://isrctn.org as ISRCTN20141297.

**Electronic supplementary material:**

The online version of this article (doi:10.1007/s10552-017-0910-2) contains supplementary material, which is available to authorized users.

## Introduction

The insulin-like growth factor (IGF) system has been implicated in the etiology and progression of various cancers, including prostate cancer [1–3]. Specifically, IGF-I is associated with increased risk of prostate cancer and higher risk of prostate cancer-specific mortality in men with advanced cancer [2, 3]. It is a potent mitogen that promotes cell proliferation, metabolism and differentiation, and inhibits apoptosis [1]. About 90% of circulating IGF-I is bound to IGF binding protein-3 (IGFBP-3) [1], which regulates the bioavailability of IGF-I and suppresses its effects by inhibiting IGF-I binding to IGF cell-surface receptors. However, epidemiological evidence on IGFBP3’s relationship with prostate cancer is mixed [2].

Circulating IGF levels are nutritionally regulated and may mediate the observed effects of diet on prostate cancer, including lycopene-rich foods, plant foods, calcium, and dairy products [4, 5]. It is well established that IGF-I is elevated by protein and energy intake in malnourished individuals [6]. Dairy and calcium intakes are positively and consistently associated with IGF-I in epidemiological studies [7, 8], with randomized clinical trials showing increased IGF-I levels with higher milk intake [9, 10]. Conversely, lycopene-rich foods [11] and plant foods [12, 13] have been inversely linked to IGF-I. Associations of the IGF system with smoking, alcohol, and physical activity are uncertain [1, 12, 14–16], while several studies have reported an inverted U-shaped relationship between body mass index (BMI) and IGF-I [17, 18].

As far as we know, no study has examined the longitudinal association of dietary and lifestyle changes with circulating IGF levels after a prostate cancer diagnosis. Most studies have been cross-sectional and involved cancer-free populations [7, 8, 12, 19]. One study investigated the 8-year associations of IGF peptides with lifestyle factors, but in young men who were cancer-free [15]. Here we investigate the association of changes in dietary intake or adherence to dietary and lifestyle recommendations with post-diagnosis circulating levels of IGF-I and IGFBP-3 in men diagnosed with prostate cancer in the Prostate testing for cancer and Treatment (ProtecT) randomized trial [20].

## Materials and methods

### Study population

ProtecT is a population-based randomized controlled trial investigating the effectiveness of treatments for PSA-detected localized prostate cancer [20]. Between 2001 and 2009, 228,966 men aged 50–69 years registered at general practices in nine UK cities were invited to attend a prostate check clinic. Over 82,000 men had a prostate-specific antigen (PSA) test, and consent was sought from men to provide additional blood samples for research purposes. They were also given a diet, health, and lifestyle (DHL) questionnaire to complete before receipt of their PSA results. Men with raised PSA (≥3 and <20 ng/mL; *n* = 8,566) were invited for repeated PSA test and a 10 core-transrectal ultrasound-guided biopsy. Tumors were assigned a Gleason score and cancers were staged using the tumor node metastasis (TNM) system.

**Fig. 1:**
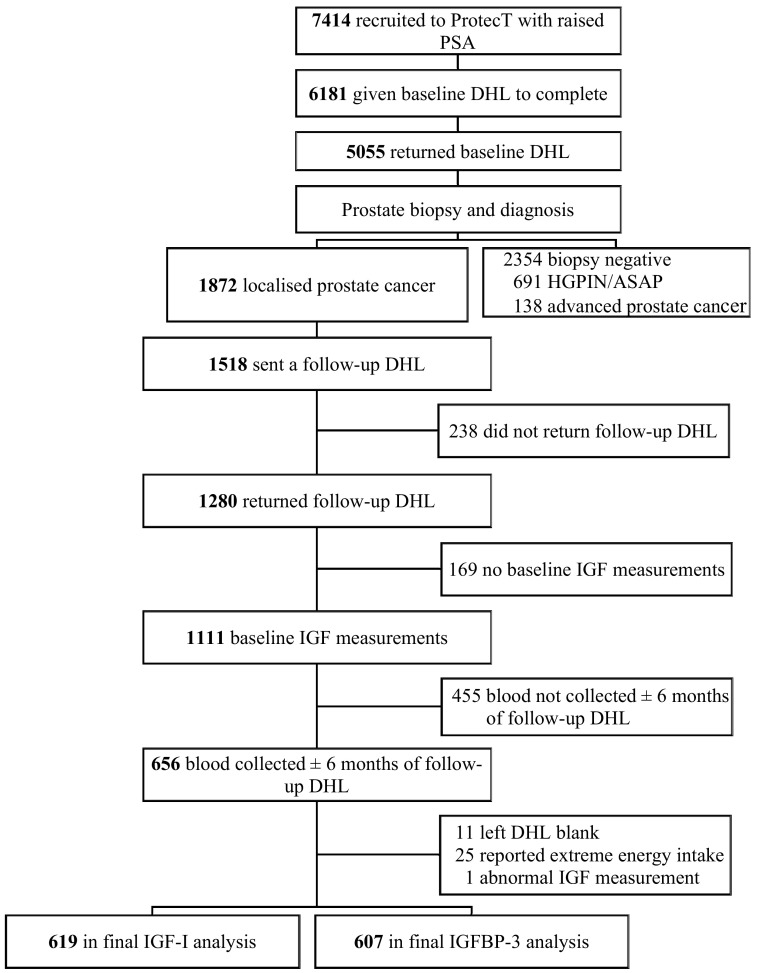
Flow diagram of participants included in analysis. *PSA* prostate-specific antigen (≥3.0 and <20.0 ng/mL). *DHL* diet, health, and lifestyle, *HGPIN* high-grade prostatic intraepithelial neoplasia, *ASAP* atypical small acinar proliferation

Of the 7,414 men who underwent biopsy, 6,181 were given a DHL questionnaire at the prostate check clinic and 5,055 returned it (Fig. 1). Overall, 1,872 men were diagnosed with localized prostate cancer (T1-T2, NX, M0), of whom 1,518 were sent a follow-up DHL questionnaire between 2007 and 2010 with a mean follow-up time of 17 months. Blood samples were also collected at annual follow-up appointments from men who provided consent. We excluded men who did not return follow-up DHL questionnaires (*n* = 238), did not have baseline serum IGF-I or IGFBP-3 measurement (*n* = 169), or did not have blood collected within ±6 months of the follow-up DHL questionnaire return date (*n* = 455). One man had a markedly raised IGF-I (617.0 ng/mL) and IGFBP-3 (8347.0 ng/mL) and was excluded. We also excluded men who left the DHL questionnaires blank (*n* = 11), or reported total energy intake <800 or >4000 kcal/day (*n* = 25) [21]. This resulted in 619 men and 607 men for the IGF-I and IGFBP-3 analyses, respectively. Study participants gave informed consent for the use of their data for research purposes. The Trent Multicentre Research Ethics Committee approved the ProtecT trial and the associated ProMPT study.

### Data collection

Trained nurses measured men’s weight at the prostate check clinic according to a standard protocol. If unavailable, self-reported weight was used (*n* = 44). Weight was self-reported only at follow-up. Height was self-reported at baseline and follow-up. BMI was derived as weight over height squared (kg/m^2^). Godin’s Leisure Time Physical Activity questionnaire was used to assess physical activity [22]. Physical activity was computed as number of times per week of moderate and strenuous exercise. Alcohol intake was estimated based on the number of units of spirits, wine, or beer consumed and the amount of alcohol (g) per drink. For smoking, we categorized men as never, former, and current smokers.

### Dietary questionnaire

Dietary intake in the past 12 months was assessed using a validated 114 item-food frequency questionnaire (FFQ) adapted from the UK arm of the EPIC study [23]. Men reported frequency of intake for each food item across nine mutually exclusive categories, ranging from “never or less than once per month” to “six or more times per day.” The assignment of portion size in grams for each food item was based on UK food portion sizes [24], food weights derived from a 7-day diet diary from a sub-sample of participants in ProtecT, and data from the Carnegie survey of diet and health [25]. Food intake was computed as the product of frequency of intake and portion size. Nutrient intake was derived by multiplying the frequency of intake by the nutrient content per portion of food, using nutrient values from the composition tables of McCance and Widdowson, and its supplements [26]. Refer to Supplementary Material 1 for definition of dairy products, fruits and vegetables, and foods rich in lycopene.

Dietary exposures were selected a priori based on their association with circulating IGFs or prostate cancer risk from the published literature and the World Cancer Research Fund and the American Institute for Cancer Research (WCRF/AICR) second expert report [4]. They include total energy intake, protein, dairy protein, dairy products, calcium, foods rich in lycopene, non-starch polysaccharide (NSP), and fruits and vegetables. Lifestyle exposures of interest were BMI, physical activity, alcohol intake and smoking status.

Since there were no recommended absolute intakes for total energy, total protein, dairy protein, and dairy products, we divided men into tertiles of baseline intake: low, medium, and high (Table 2), and categorized follow-up intake using the same cut-offs. We grouped men into three ‘change’ categories: no/minimal change (i.e., same category at baseline and follow-up assessments), decreased intake (high to low; or high to medium; or medium to low), and increased intake (low to medium; low to high; or medium to high). Where dietary or lifestyle public health recommendations were available, we categorized men into two categories based on their level of adherence (adherent vs. non-adherent) (Table 2). The cut-off criteria were derived from the WCRF/AICR second expert report for calcium, BMI, physical activity, and alcohol [4], and the Health Professionals Study for tomato and tomato products [27]. Cut-offs for non-starch polysaccharides [28] and fruits and vegetables [29] concur with UK dietary guidelines. We grouped men into four categories: non-adherent (NAd, i.e., non-adherent before and after diagnosis), non-adherent to adherent (NAd→Ad, i.e., men who became more ‘healthy’ after diagnosis), adherent to non-adherent (Ad→NAd, i.e., men who became less ‘healthy’ after diagnosis), and adherent (Ad, i.e., adherent before and after diagnosis).

### Blood collection and IGF assays

Non-fasted blood samples were drawn from men at recruitment (pre-diagnosis) between 2003 and 2008, and at annual follow-up appointments between 2007 and 2010. The samples were left to stand at room temperature and then centrifuged at approximately for 20 min to extract serum. They were kept at 5 °C during transportation to a laboratory, where they were aliquoted for storage at −80 °C within 36 h of collection. Baseline and follow-up IGF-I and IGFBP-3 assays were carried out in JMPH’s laboratory by staff blinded to dietary and lifestyle data using an in-house radio-immunoassay [30, 31], which measures total IGF-I and IGFBP-3, including all forms that have undergone minor fragmentation. Measurements were performed in triplicates and an average was computed for analyses.

Baseline serum samples were assayed between 2007 and 2010 to investigate the associations of IGFs and IGFBPs with prostate cancer risk in a case–control study nested within ProtecT [32]. Mean intra-assay coefficients of variation for baseline IGF-I and IGFBP-3 were 7.4 and 8.9%, and mean inter-assay coefficients of variation were 11.3 and 12.5%. All follow-up serum samples were assayed in 2014. Mean intra-assay coefficients of variation for follow-up IGF-I and IGFBP-3 were 7.6 and 6.8%, and mean inter-assay coefficients of variation were 10.0 and 10.5%.

### Statistical analysis

Serum IGF-I and IGFBP-3 were approximately normally distributed. We used analysis of covariance (ANCOVA) to estimate associations of dietary and lifestyle changes with post-diagnosis IGF-I and IGFBP-3 separately (see Supplementary Material 1) [33, 34]. All models were adjusted for baseline IGF values, baseline dietary, or lifestyle exposure of interest, baseline age, and follow-up time; for dietary exposures, the models were also adjusted for the difference in baseline and follow-up total energy intake (kcal/day) (Model 1 in Tables 3, 4, 5).

We compared the basic ANCOVA models with the models additionally adjusted for the following confounding factors identified a priori: height (m), self-reported diabetes, occupational class, prostate cancer treatment received, and cancer grade. For the purpose of this analysis, tumors with Gleason score of ≤6 were defined as low, and ≥7 as high, grade. For dietary exposures, we also assessed potential confounding by baseline smoking status, dietary supplement intake, change in BMI, physical activity, and alcohol intake. However, most of these additional variables did not confound any of the observed associations, and only treatment received and cancer grade were added (in addition to the variables listed above in Model 1) to the fully adjusted regression models (Model 2 in Tables 3, 4, 5). Estimates from the fully adjusted regression models were presented as mean percentage difference of follow-up IGF-I or IGFBP-3, and are used to predict mean post-diagnosis IGF-I or IGFBP-3 levels for each category of pre- to post-diagnosis change in dietary or lifestyle behaviors. We estimated the Bonferroni correction for multiple testing based on the 24 tests carried out. All statistical analyses were performed using Stata v12.1 (StataCorp, College Station, TX USA).

## Results

Our study population was predominantly White with an average age of 62 years, and a mean BMI of 27.1 kg/m^2^ at recruitment (Table 1). The mean IGF-I and IGFBP-3 levels were 22.0 nmol/mL (standard deviation (SD): 7.1 nmol/mL) and 160.2 nmol/mL (SD: 34.5 nmol/mL), respectively. The majority of the men were diagnosed with low grade cancer (72.7%). Only a small proportion of men had a family history of prostate cancer (8.7%) or had diabetes (5.3%), and 52.3% of the men in the study reported taking dietary supplements.

**Table 1:** Baseline characteristics of participants

Characteristics	*n* = 619	Mean (SD) or %
Age at recruitment (years)	619	62.0 (4.9)
Height (m)	610	1.76 (0.06)
Weight (kg)	618	84.2 (12.0)
BMI (kg/m^2^)	610	27.1 (3.5)
Time since diagnosis (months)	619	17 (9)
Serum IGF concentrations (nmol/mL)		
IGF-I	619	22.0 (7.1)
IGFBP-3	607	160.2 (34.5)
Ethnicity		
White	607	98.1
Others	5	0.8
Unknown	7	1.1
Occupational class		
Managerial	273	44.1
Intermediate	99	16.0
Working	238	38.5
Unknown	9	1.4
Family history of prostate cancer		
Yes	54	8.7
No	512	82.7
Do not know	41	6.6
Unknown	12	2.0
Diabetes		
Yes	33	5.3
No	541	87.4
Unknown	45	7.3
PSA level		
<10.0 ng/mL	549	88.7
10.0–20.0 ng/mL	70	11.3
Treatment		
Active monitoring	275	44.4
Prostatectomy	176	38.4
Radiotherapy	167	27.0
Other	1	0.2
Gleason grade^a^		
Low (2–6)	450	72.7
High (7–10)	169	27.3
Vitamin/dietary supplement intake		
Yes	324	52.3
No	282	45.6
Unknown	13	2.1

^a^Gleason scores of 2, 3, and 4 were acceptable when the ProtecT trial was conducted as it was before the 2005 International Society of Urological Pathology [44]

Table 2 shows the cut-off criteria for categorizing dietary intake and adherence to dietary and lifestyle recommendations pre- and post-diagnosis, along with the proportion of men in each category. Intake of total energy, non-starch polysaccharide, and fruits and vegetables remained largely the same before and after diagnosis. There was a decrease in protein and dairy product intake post-diagnosis: the proportion of men who had high protein intake decreased by 3.9%, and there were 4.5% fewer men with a high intake of dairy products. The reduction in protein intake may in part be due to a smaller contribution of dairy-derived protein (4.9% decrease in high intake of dairy protein post-diagnosis). Conversely, men increased their consumption of foods rich in lycopene following a diagnosis. There was a 2.5% increase in men who had over 10 servings of tomatoes and tomato products per week; adherence to the physical activity recommendation also increased by about 3% following a diagnosis. There was a 2.2% fall in the proportion of overweight or obese men. Some men (2%) quit smoking following a prostate cancer diagnosis, but alcohol consumption largely remained unchanged.

**Table 2:** Dietary intake and adherence to public health recommendations before and after diagnosis

	Cut-off points	Intake/adherence^a^	Pre-diagnosis (*n* = 619)		Post-diagnosis (*n* = 619)		p-value^f^
			*n*	%	*n*	%	
Total energy	800.0 to <1995.3 kcal/day	Low	207	33.4	210	33.9	
	≥1995.3 to <2529.8 kcal/day	Medium	206	33.3	208	33.6	
	≥2529.8 to 4000.0 kcal/day	High	206	33.3	201	32.5	0.78
Total protein	<76.3 g/day	Low	207	33.4	215	34.7	
	≥76.3 to < 97.9 g/day	Medium	206	33.3	222	35.9	
	≥97.9 g/day	High	206	33.3	182	29.4	0.27
Dairy protein	<12.1 g/day	Low	207	33.4	240	38.8	
	≥12.1 g to <18.6 g/day	Medium	206	33.3	203	32.8	
	≥18.6 g/day	High	206	33.3	176	28.4	0.03
Dairy products^b^	<292.7 g/day	Low	207	33.4	233	37.6	
	≥293.7 to < 439.4 g/day	Medium	207	33.4	209	33.8	
	≥439.4 g/day	High	205	33.1	177	28.6	0.06
Calcium	≥700 to < 1500 mg/day	Ad	481	77.7	464	75.0	
	<700 or ≥ 1500 mg/day	Non-Ad	138	22.3	155	25.0	0.18
Tomato products^b,c^	>10 servings/week	Ad	56	9.0	71	11.5	
	≤10 servings/week	Non-Ad	563	91.0	548	88.5	0.09
Non-starch polysaccharides	≥18 g/day	Ad	380	61.4	385	62.2	
	<18 g/day	Non-Ad	239	38.6	234	37.8	0.70
Fruits and vegetables^d,e^	≥5 portions/day	Ad	355	57.4	353	57.0	
	<5 portions/day	Non-Ad	264	42.6	266	43.0	0.87
Body mass index^e^	≥18.5 to <25 kg/m^2^	Ad	167	28.5	180	30.7	
	≥25 kg/m^2^	Non-Ad	419	71.5	406	69.3	0.09
Physical activity^b,e^	≥7 times/week	Ad	165	29.1	183	32.3	
	<7 times/week	Non-Ad	402	70.9	384	67.7	0.16
Alcohol^e^	≤20 g/day	Ad	351	57.5	353	57.9	
	>20 g/day	Non-Ad	259	42.5	257	42.1	0.86
Smoking status^e^	Never	n/a	256	42.1	255	41.9	
	Former	n/a	295	48.4	307	50.4	
	Current	n/a	58	9.5	47	7.7	0.09

^a^ *Ad* Adherence, *Non-Ad* Non-adherence

^b^For definition of dairy products, tomato products, and physical activity, refer to Methods and Supplementary Material 1

^c^Includes fresh tomatoes

^d^1 portion equivalent to 400 g

^e^Men with complete data only: BMI (*n* = 586), physical activity (*n* = 567), alcohol (*n* = 610), smoking status (*n* = 609)

^f^ *p*-Values obtained from McNemar test for binary variables, and likelihood ratio test for 3-level variables

Table 3 presents the associations of changes in total energy, total protein, dairy protein, and dairy product intake with IGF-I and IGFBP-3 levels following a prostate cancer diagnosis. Average IGF-I levels were 6.5% (95% CI −12.8, −0.3%, *p* = 0.04) lower in men who decreased their protein intake compared to men who did not change (minimal change). None of the dietary exposures we investigated were associated with post-diagnosis IGFBP-3.

**Table 3:** Changes in dietary intake and follow-up IGF level, adjusted for baseline IGF

	*n*	Mean change in intake	Mean follow-up intake	Mean follow-up IGF^a^	Difference (95% CI) in mean follow-up serum IGF concentration (%)	
					Model 1^b^	Model 2^c^
IGF-I (*n* = 619)						
Total energy (kcal/day)						
No change	309	−13.5	2294.8	20.3	Ref	Ref
Decreased	156	−649.3	1955.1	20.3	−1.2 (−7.0, 4.6)	−1.7 (−7.5, 4.1)
Increased	154	661.3	2602.7	20.8	4.3 (-1.5, 10.2)	4.3 (-1.6, 10.1)
Total protein (g/day)						
No change	325	0.0	86.6	20.8	Ref	Ref
Decreased	158	−26.6	75.8	19.5	−6.5 (−12.9, −0.2)	−6.5 (−12.8, −0.3)*
Increased	136	25.4	102.2	20.5	3.7 (−2.9, 10.2)	4.5 (−2.0, 11.1)
Dairy protein (g/day)						
No change	351	−0.3	15.5	20.2	Ref	Ref
Decreased	160	−7.4	11.9	21.0	3.3 (−2.3, 9.0)	4.1 (−1.5, 9.7)
Increased	108	7.0	19.1	20.1	−0.2 (−6.7, 6.4)	0.2 (−6.3, 6.7)
Dairy products^d^ (g/day)						
No change	372	−10.2	360.1	20.5	Ref	Ref
Decreased	148	−181.2	274.7	19.8	−2.3 (−8.1, 3.4)	−2.2 (−7.8, 3.5)
Increased	99	179.3	462.3	20.7	2.5 (−4.2, 9.1)	2.0 (−4.6, 8.6)
IGFBP-3 (*n* = 607)						
Total energy (kcal/day)						
No change	301	−10.0	2304.7	130.6	Ref	Ref
Decreased	154	−648.4	1952.4	133.9	2.7 (−2.7, 8.2)	2.8 (−2.7, 8.2)
Increased	152	660.8	2601.1	130.3	0.4 (−5.1, 5.9)	0.6 (−5.0, 6.1)
Total protein (g/day)						
No change	320	0.2	86.8	133.0	Ref	Ref
Decreased	157	−26.4	75.9	129.4	−2.2 (−8.2, 3.7)	−2.3 (−8.3, 3.7)
Increased	134	25.6	102.1	129.7	−2.2 (−8.4, 4.0)	−2.0 (−8.3, 4.2)
Dairy protein (g/day)						
No change	344	−0.3	15.4	130.1	Ref	Ref
Decreased	156	−7.4	12.0	131.5	1.8 (−3.6, 7.1)	2.0 (−3.3, 7.4)
Increased	107	7.0	19.1	135.3	3.1 (−3.0, 9.2)	3.0 (−3.1, 9.2)
Dairy products^d^ (g/day)						
No change	367	−11.0	360.7	130.7	Ref	Ref
Decreased	143	−183.8	274.3	128.3	−0.4 (−5.8, 5.0)	−0.3 (−5.7, 5.1)
Increased	97	180.0	461.9	138.4	5.3 (−0.9, 11.5)	5.2 (−1.1, 11.4)

^a^Mean predicted from fully adjusted regression model (nmol/mL)

^b^Adjusted for baseline age, baseline IGF, baseline dietary intake, follow-up time point, and changes in energy intake (except that for total energy)

^c^Further adjusted for treatment received and cancer grade

^d^For definition of dairy products, refer to Supplementary Material 1. * *p* = 0.04

Table 4 shows the associations of changes in adherence to dietary recommendations with IGF-I and IGFBP-3 levels following a prostate cancer diagnosis. Men who consumed <5 portions/day of fruits and vegetables before diagnosis, but increased to ≥5 after their diagnosis (NAd→Ad), had post-diagnosis IGF-I levels that were on average 12.0% (95% CI −20.1, −3.9%; *p* = 0.002) lower than those who did not change and consumed <5 portions/day (NAd). Average post-diagnosis IGF-I levels were also lower among men who had ≥5 portions/day of fruits and vegetables before diagnosis but decreased to <5 after diagnosis (Ad→NAd: −10.1%, 95% CI −18.4, −1.8%, *p* = 0.02). Adherence to the fruits and vegetables recommendation before and after diagnosis (Ad) was also linked to post-diagnosis IGF-I levels that were 8.8% lower on average (95% CI −15.8, −1.8, *p* = 0.01). Conversely, post-diagnosis serum IGFBP-3 levels were not associated with changes in adherence to recommendations on calcium, tomatoes and tomato products, non-starch polysaccharide, or fruits and vegetables.

**Table 4:** Changes in adherence to dietary recommendations and follow-up IGF level, adjusted for baseline IGF

	*n*	Mean change in intake	Mean follow-up intake	Mean follow-up IGF^a^	Difference (95% CI) in mean follow-up serum IGF concentration (%)	
					Model 1^b^	Model 2^c^
IGF-I (n = 619)						
Calcium (mg/day)						
Non-adherent	66	19.5	1038.4	20.0	Ref	Ref
Ad→NAd	89	−95.5	944.9	20.7	0.1 (−9.4, 9.5)	−0.4 (−9.8, 8.9)
NAd→Ad	72	−23.6	1049.9	19.8	−0.6 (−10.5, 9.2)	−0.3 (−10.0, 9.5)
Adherent	392	−19.3	1025.4	20.5	0.7 (−7.0, 8.4)	0.5 (−7.2, 8.1)
Tomato products^d,e^ (serving/week)						
Non-adherent	517	0	4.5	20.7	Ref	Ref
Ad→NAd	31	−9.5	6.0	18.5	−14.1 (−28.4, 0.1)	−13.8 (−27.9, 0.3)
NAd→Ad	46	9.5	16.0	19.0	−6.3 (−15.3, 2.7)	−5.3 (−14.2, 3.7)
Adherent	25	−2.5	15.0	19.9	−3.7 (−19.6, 12.3)	−6.2 (−22.1, 9.6)
NSP (g/day)						
Non-adherent	153	0.2	13.7	21.1	Ref	Ref
Ad→NAd	81	−8.7	15.2	19.4	−4.9 (−13.9, 4.2)	−4.6 (−13.5, 4.4)
NAd→Ad	86	8.4	23.0	19.4	−3.9 (−12.3, 4.5)	−3.6 (−11.9, 4.8)
Adherent	399	0.3	27.1	20.6	−0.7 (−8.8, 7.5)	−0.4 (−8.5, 7.6)
Fruit and vegetables^d^ (portion/day)						
Non-adherent	193	0	3.5	21.6	Ref	Ref
Ad→NAd	73	−2.5	4.0	20.2	−10.4 (−18.9, −2.0)	−10.1 (−18.4, −1.8)*
NAd→Ad	71	3.0	7.0	18.3	−12.9 (−21.0, −4.7)	−12.0 (−20.1, −3.9)***
Adherent	282	0	8.0	20.2	−8.9 (−15.9, −1.8)	−8.8 (−15.8, −1.8)**
IGFBP-3 (*n* = 607)						
Calcium (mg/day)						
Non-adherent	64	22.5	1056.4	124.7	Ref	Ref
Ad→NAd	86	−97.8	943.8	132.6	3.7 (−5.2, 12.6)	3.9 (−5.0, 12.9)
NAd→Ad	72	−23.6	1049.9	126.7	1.3 (−7.9, 10.5)	1.1 (−8.1, 10.4)
Adherent	385	−18.6	1026.3	133.1	5.1 (−2.2, 12.3)	5.0 (−2.2, 12.3)
Tomato products^d,e^ (serving/week)						
Non-adherent	506	0	4.5	132.4	Ref	Ref
Ad→NAd	31	−9.5	6.0	137.9	2.0 (−11.3, 15.2)	1.9 (−11.4, 15.2)
NAd→Ad	45	9.5	16.0	124.4	−5.3 (−13.8, 3.1)	−5.2 (−13.7, 3.2)
Adherent	25	−2.5	15.0	114.1	−12.5 (−27.4, 2.3)	−13.5 (−28.4, 1.5)
NSP (g/day)						
Non-adherent	151	0.2	13.7	134.2	Ref	Ref
Ad→NAd	77	−8.7	15.3	129.4	−0.6 (−9.1, 8.0)	−0.7 (−9.3, 7.8)
NAd→Ad	85	8.3	22.9	128.2	−2.2 (−10.0, 5.6)	−2.0 (−9.9, 5.9)
Adherent	294	0.3	27.1	131.3	0.0 (−7.6, 7.7)	0.0 (−7.6, 7.7)
Fruit and vegetables^d^ (portion/day)						
Non-adherent	189	0	3.5	131.6	Ref	Ref
Ad→NAd	71	−2.5	4.0	136.2	3.9 (−4.1, 11.9)	4.2 (−3.8, 12.2)
NAd→Ad	69	3.0	7.0	123.7	−2.7 (−10.4, 5.0)	−2.3 (−10.1, 5.5)
Adherent	278	0	8.0	131.9	1.4 (−5.2, 8.0)	1.4 (−5.2, 8.1)

^a^Mean predicted from fully adjusted regression model

^b^Adjusted for baseline age, baseline IGF, baseline dietary intake, follow-up time point, and changes in energy intake

^c^Further adjusted for treatment received and cancer grade

^d^Rounded to the nearest 0.5 serving/week or portion/day

^e^Includes fresh tomatoes. For definition of tomato products, refer to Supplementary Material 1. *NSP* non-starch polysaccharides

*p = 0.02, ** *p* = 0.01, *** *p* = 0.002

Table 5 presents the associations of lifestyle changes with IGF-I and IGFBP-3 levels following a prostate cancer diagnosis. There was weak evidence that men who were active before diagnosis but became inactive after their diagnosis (Ad→NAd) had post-diagnosis IGF-I levels that were 9.5% higher on average (95% CI 0.1, 18.9%, *p* = 0.05) than men who were inactive and did not change. Average post-diagnosis IGFBP-3 levels were 14.6% (95% CI −24.5, −4.8%; *p* = 0.004) lower in men who were overweight before diagnosis and acquired a healthy BMI after diagnosis (NAd→Ad). A similar difference was observed for men who had healthy BMI before and after diagnosis (Ad vs NAd: −9.2%, 95% CI −16.8, −1.6%; *p* = 0.02). Post-diagnosis IGFBP-3 levels were 10.7% (95% CI −19.3, −2.1%, *p* = 0.02) lower in men who adhered to the physical activity recommendation before and after diagnosis compared to men who were non-adherent and did not change. Finally, the association between increased fruit and vegetable intake and IGF-I level post-diagnosis was the only finding robust to Bonferroni correction (i.e., *p* = 0.05/24; *p* = 0.002).

**Table 5:** Changes in adherence to lifestyle recommendations and follow-up IGF level, adjusted for baseline IGF

	*n*	Mean change	Mean at follow-up	Mean follow-up IGF^a^	Difference (95% CI) in mean follow-up serum IGF concentration (%)^b^	
					Model 1^b^	Model 2^c^
IGF-I (*n* = 619)						
BMI (kg/m^2^)						
Non-adherent	383	−0.2	28.6	20.3	Ref	Ref
Ad→NAd	23	1.4	25.7	21.7	3.1 (−10.1, 16.2)	6.6 (−6.6, 19.8)
NAd→Ad	36	−1.8	24.2	20.6	−1.3 (−11.6, 9.1)	0.4 (−9.9, 10.6)
Adherent	144	−0.1	22.9	20.1	−5.7 (−13.7, 2.3)	−4.2 (−12.1, 3.8)
Physical activity^d^ (times/week)						
Non-adherent	321	0.5	2.5	20.5	Ref	Ref
Ad→NAd	65	−5.0	3.5	21.3	10.1 (0.6, 19.7)	9.5 (0.1, 18.9)*
NAd→Ad	83	5.5	8.5	21.2	3.8 (−3.4, 11.0)	3.3 (−3.9, 10.4)
Adherent	100	0.5	10.5	19.6	3.7 (−5.7, 13.1)	4.2 (−5.1, 13.5)
Alcohol intake (g/day)						
Non-adherent	199	1.1	45.9	20.2	Ref	Ref
Ad→NAd	58	15.6	29.1	19.7	0.4 (−9.7, 10.5)	2.1 (−7.9, 12.1)
NAd→Ad	60	−22.5	11.9	20.3	3.2 (−5.5, 11.8)	5.8 (−2.8, 14.5)
Adherent	293	−0.7	6.3	20.7	6.6 (−1.7, 14.9)	8.0 (−0.2, 16.2)
Smoking^d^						
Never	256	n/a	n/a	20.7	Ref	Ref
Former	295	n/a	n/a	20.1	−1.1 (−6.1, 3.8)	−1.1 (−6.0, 3.8)
Current	58	n/a	n/a	20.9	−0.2 (−8.5, 8.1)	−0.2 (−8.4, 8.1)
IGFBP-3 (*n* = 607)						
BMI (kg/m^2^)						
Non-adherent	377	−0.2	28.6	133.4	Ref	Ref
Ad→NAd	22	1.4	25.6	131.4	−7.7 (−20.2, 4.8)	−8.2 (−21.0, 4.6)
NAd→Ad	35	−1.8	24.2	123.6	−14.9 (−24.7, −5.1)	−14.6 (−24.5, −4.8)***
Adherent	140	−0.1	23.0	126.1	−9.5 (−17.0, −2.0)	−9.2 (−16.8, −1.6)**
Physical activity^d^ (times/week)						
Non-adherent	311	0	2.5	130.2	Ref	Ref
Ad→NAd	64	−5.0	3.5	143.5	3.5 (−5.2, 12.3)	3.5 (−5.2, 12.3)
NAd→Ad	82	5.5	8.5	129.7	−2.4 (−9.0, 4.2)	−2.6 (−9.2, 4.0)
Adherent	98	0.5	10.5	126.6	−10.7 (−19.3, −2.1)	−10.7 (−19.3, −2.1)**
Alcohol intake (g/day)						
Non-adherent	198	0.9	45.5	133.8	Ref	Ref
Ad→NAd	57	15.8	29.2	134.8	2.3 (−7.2, 11.7)	2.8 (−6.7, 12.2)
NAd→Ad	60	−22.5	11.9	129.8	−1.1 (−9.2, 6.9)	−0.4 (−8.5, 7.7)
Adherent	283	−0.7	6.3	129.3	−2.2 (−9.9, 5.5)	−2.0 (−9.7, 5.8)
Smoking^e^						
Never	255	n/a	n/a	136.6	Ref	Ref
Former	296	n/a	n/a	130.1	−0.5 (−5.1, 4.1)	−0.5 (−5.1, 4.1)
Current	56	n/a	n/a	132.1	−0.0 (−7.9, 7.9)	−0.5 (−8.5, 7.6)

^a^Mean predicted from fully adjusted regression model

^b^Adjusted for baseline age, baseline IGF, baseline lifestyle exposure, follow-up time point

^c^Further adjusted for treatment received and cancer grade

^d^Rounded to the nearest 0.5 times/week

^e^Baseline smoking status only was evaluated due to minimal change at follow-up. *n/a* not applicable

*p = 0.05, ** *p* = 0.02 *** *p* = 0.004

## Discussion

To our knowledge, our study is the first to assess associations of changes in dietary and lifestyle behaviors with circulating IGF-I and IGFBP-3 levels following a diagnosis of prostate cancer. We observed associations of post-diagnosis serum IGF-I and IGFBP-3 levels with changes in protein intake and changes in adherence to recommendations on fruits and vegetables, BMI, and physical activity following a prostate cancer diagnosis.

Unlike most studies [35–37], there were negligible changes in smoking, non-starch polysaccharide, and fruit and vegetable intake in our study population. Instead, there was a small reduction in the proportion of men in the high dairy intake category (median decrease of 0.5 serving/day in men who decreased intake) and a slight increase in the proportion of men who consumed >10 servings/week of tomatoes and tomato products (median increase of 8.0 servings/week in men who increased intake). This could be due to a heightened awareness of the link between these dietary factors and prostate cancer risk since the publication of the WCRF/AICR second expert report [4]. There was also a marginal increase in the proportion of men who adhered to the physical activity or healthy weight recommendation post-diagnosis (mean increase of 5.5 times/week of moderate to strenuous physical activity, and mean reduction of 1.8 kg/m^2^ in BMI), which is in line with the findings from several studies [37, 38].

Our finding of a lower average post-diagnosis IGF-I level in men who reduced their protein intake is supported by most studies, which found a positive link between protein intake and IGF-I [6–8]. The decrease in protein intake in our study may be attributed to a lower intake of dairy products and decreased protein intake from dairy sources. It has been postulated that the protein fraction of dairy (i.e., dairy protein) drives the positive relationship between IGF-I and dairy intake [8, 10, 39]. However, we did not observe any associations of changes in dairy product or dairy protein intake with post-diagnosis IGF-I or IGFBP-3.

Men who adhered to the recommendation on fruits and vegetables (≥5 portions/day) post-diagnosis had lower average IGF-I levels than men who were non-adherent and did not change. Similarly, men who were adherent to the recommendation pre- and post-diagnosis had lower IGF-I levels. High vegetable intake has been linked to lower circulating levels of IGF-I or higher IGFBP-3 in some [12, 13, 19] but not all studies [39, 40]. However, IGF-I levels were also lower in men who decreased their intake to fewer than five portions a day after a diagnosis in our study. It is possible that pre-diagnosis diet reflects long-term dietary intake, so long-term adherence to the recommendation on fruits and vegetables may offset the impact of lower fruits and vegetables intake after a diagnosis on circulating IGF-I. Nonetheless, we cannot exclude the possibility of a chance findings in the context of potential misclassification of exposure errors, limited power, and multiple testing.

We did not find any links between changes in lifestyle factors and post-diagnosis IGF-I, except for physical activity. Men who were active before diagnosis but became inactive had higher average post-diagnosis IGF-I levels, but the strength of evidence was weak. Post-diagnosis IGFBP-3 was lower in men who met the BMI recommendation (<25.0 kg/m^2^), especially among men who were overweight before diagnosis but acquired a healthy BMI after diagnosis. The positive association between BMI and IGFBP-3 was previously observed in cancer-free men in the ProtecT study [17]. IGFBP-3 increased by 63.5 ng/ml per SD higher in BMI (95% CI −2.69–129.8, *p* = 0.06). Conversely, the Coronary Artery Risk Development in Young Adults (CARDIA) study found no association between 8-year changes in BMI and IGFBP-3 [15].

Strengths of our study include its size and population-based, prospective design. Detection bias was minimized, as case finding was part of the trial design. There were standardized records of cancer stage and grade, and types of treatment. We also have blood samples collected close to the return date of the follow-up questionnaires (±6 months). However, most participants were White European, and there are some ethnic differences in dietary and lifestyle associations with IGF peptides [15, 19, 41], so our findings may not be generalizable to other ethnic groups.

Although we used validated and detailed questionnaires to minimize measurement error, there will still be some misclassification of exposures. Compared to food diaries, FFQ is prone to a greater degree of misclassification [42, 43], but the effect is likely to be non-differential in our study as baseline questionnaires were completed by 54.1% of men before receipt of initial PSA test results, and men were not given any dietary advice after diagnosis. Therefore, true associations of dietary and lifestyle changes with post-diagnosis circulating IGF-I and IGFBP-3 might be underestimated.

There was variation in baseline and follow-up serum sample storage time, ranging from 0 to 7 years. Nonetheless, storage time was not associated with baseline or follow-up circulating IGF-I and IGFBP-3 in univariable analyses. Finally, using a conservative Bonferroni multiple testing penalty would lead to just one strong finding. However, our study is the first to find such differences in IGF-I and IGFBP-3 post-diagnosis, and other studies are needed to replicate our novel findings. To minimize multiple testing, we had decided a priori on the dietary and lifestyle variables to be tested and used established dietary guidelines and lifestyle recommendations for categorization where available.

In conclusion, decreased protein intake and BMI, and increased fruits and vegetables intake and physical activity, following a prostate cancer diagnosis were associated with reduced post-diagnosis serum IGF-I and IGFBP-3. As one of the first studies to identify these links, our findings warrant confirmation in other studies and may inform future dietary and lifestyle interventions in men with prostate cancer.

## Electronic supplementary material

Below is the link to the electronic supplementary material.

Supplementary material 1 (DOCX 39 kb)
