# Supplementary material for: Post-diagnosis serum insulin-like growth factors in relation to dietary and lifestyle changes in the Prostate testing for cancer and Treatment (ProtecT) trial
Source: Cancer Causes Control. 2017 Jun 23;28(8):877–88. doi: 10.1007/s10552-017-0910-2 (PMC5501895; doi:10.1007/s10552-017-0910-2)

Supplementary Material 1

**Definition of dairy products, fruits and vegetables and foods rich in lycopene**

*Dairy products.* Dairy products (portion size) include single or sour cream (30g), double or clotted cream (60g), yoghurt (125g), cheese (40g), cottage cheese or low fat soft cheese (89g), and milk, including full cream, semi skimmed, skimmed, Channel Island and dried milk (200mL).

*Fruits and vegetables*. We only included whole fruit and vegetable intake, and computed daily intake in grams. One portion is equivalent to 80g. Potatoes, fruit and vegetable juices were excluded.

*Foods rich in lycopene.* Fresh tomato and tomato product intake was used as an indicator of lycopene intake as they are rich sources of lycopene. Tomato products include tomato juice (160mL), tomato sauce (12g), pizza (300g) and baked beans (135g).

Equation 1: Basic ANCOVA model

Post diagnosis IGF= constant + β_1_ dietary or lifestyle changes (categorical) + β_2_ baseline IGF +

β_3_ baseline age + β_4_ baseline diet or lifestyle + β_5_ follow-up time-point +

error term

Thus, the slope, β_1_ is defined as the difference in mean post-diagnosis IGF levels comparing individuals in the exposed group to the reference group, conditioned on baseline IGF, age, diet or lifestyle exposure of interest and follow-up time-point. The reference group was ‘no change’ or ‘non-adherent’.

Using IGF change as the outcome variable is arguably more intuitive, but is not appropriate here as it does not control for ‘regression to the mean’: baseline IGF is negatively correlated with change in IGF because individuals with unusually low baseline IGF values tend to increase, while those with unusually high baseline IGF values tend to decrease, when the values are re-measured at a later time-point (33) (**Supplementary Figure 1**).

**Supplementary Figure 1. Regression to the mean. Baseline IGF-1 (x-axis) vs Change in IGF-1 (y-axis)**


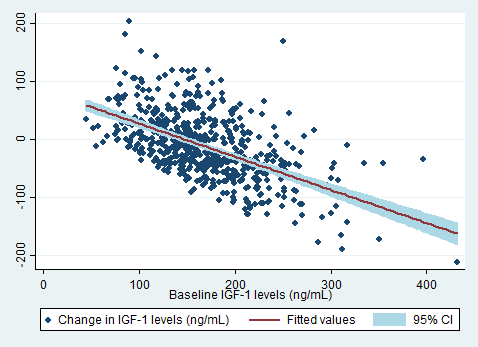

Supplement: Supplementary file 1 — Supplementary material 1 (DOCX 39 kb) [file 10552_2017_910_MOESM1_ESM.docx]
